# Comparative Analysis of Milk Fat Globular Membrane (MFGM) Proteome between Saudi Arabia *Camelus dromedary* Safra and Wadha Breeds

**DOI:** 10.3390/molecules25092146

**Published:** 2020-05-04

**Authors:** Bassam H. Sabha, Afshan Masood, Ibrahim O. Alanazi, Assim A. Alfadda, Hussein A. Almehdar, Hicham Benabdelkamel, Elrashdy M. Redwan

**Affiliations:** 1 Department of Biological Science, Faculty of Science, King Abdulaziz University, Jeddah, P.O. Box 80203, Jeddah 21589, Saudi Arabia; bassam_alsobahy@hotmail.com (B.H.S.); dralmehdar@hotmail.com (H.A.A.); 2 Proteomics Unit, Obesity Research Center, College of Medicine, King Saud University, P.O. Box 2925 (98), Riyadh 11461, Saudi Arabia; afsmasood@ksu.edu.sa (A.M.); aalfadda@ksu.edu.sa (A.A.A.); 3 The National Center for Genomic Technology (NCGT), Life Science and Environment Research Institute, King Abdulaziz City for Science and Technology (KACST), PO Box 6086, Riyadh 11461, Saudi Arabia; ialenazi@kacst.edu.sa

**Keywords:** Safra breed, Wadha breed, camel milk, proteomics, 2D-DIGE, MFGM

## Abstract

Camel milk is traditionally known to have medicinal properties and many potential health benefits. Natural milk contains many soluble proteins and nanoparticles, such as a milk fat globule membrane (MFGM), a three-layered membrane covering of milk fat globule mainly composed of proteins and lipids, which plays an important role in human health. MFGM proteins account for 1%–4% of total milk proteins, and their nutritive value and distribution depends on the different breeds. The differential composition of these membrane proteins among different camel breeds has not been explored. The current study, therefore, aimed to quantitatively analyze and compare the MFGM proteome between the milk produced by the two most common Saudi camel breeds, *Camelus dromedarius*: Safra and Wadha. Two-dimensional difference in gel electrophoresis (2D-DIGE) and mass spectrometry analysis revealed a total of 44 MFGM proteins that were identified with a significant difference in abundance (*p* ≤ 0.05; fold change ≥ 1.5) between the two breeds. Thirty-one proteins were up-regulated and 13 proteins were down-regulated in the Safra breed compared to the Wadha breed. The proteins identified with an increased abundance included α-lactalbumin, lactadherin, and annexin a8, whereas the down-regulated proteins included butyrophilin subfamily 1 member a1, lactotransferrin, and vinculin. The differentially abundant proteins were analyzed by the UNIPROT system and gene ontology (GO) to reveal their associations with known biological functions and pathways. Enzyme-linked immunosorbent assay (ELISA) confirmed the 2D-DIGE findings of butyrophilin (BTN) and α-lactalbumin (α-LA) levels obtained from Safra and Wadha breeds.

## 1. Introduction

Camel milk has been consumed for thousands of years because of its immense nutritional value and health benefits. Several studies have revealed that camel milk has a high concentration of immunoglobulins, serum albumin, lactoferrin, the hormone insulin, and other important ingredients [1,2,3]. Recently, camel milk has been utilized as medication for treating many diseases including gastrointestinal disorders, diabetes mellitus, food allergy, cancer, and hepatitis C and B [1,4,5,6,7]. Moreover, camel milk has differential in vitro antimicrobial activities against many pathogens [6,7], based on its bioactive proteins and/or peptides [1,8,9,10,11,12].

The characteristics and composition of camel milk are known to differ due to various factors, including the breed of the camel, conditions of feeding, the health of the animal, calving number, stage of lactation, geographical location, and with seasons [13,14]. Milk fat globule membrane (MFGM) is one of the key components of not only camel milk but in the milk of other primates as well. It is a three-layered membrane covering the milk fat globule membrane, accounting for 1%–4% of total milk proteins [15]. The origin of MFGM has been studied to be from different parts of cells including the endoplasmic reticulum membrane, the apical plasma membrane of secretory cells in the mammary gland and cytoplasm [16]. It encloses the lipid molecules in the milk, which mainly comprises triacylglycerides, phospholipids, cholesterol, and various other integral, peripheral proteins, and other compositions derived from the original mammary cell [17]. MFGM derived proteins have been involved in beneficial bioactivities, such as antibacterial, antiviral [18], anticancer effects [19], and anti-inflammation [20].

Today, the benefits of MFGM have transferred it from the bedside into the market [21,22,23,24,25,26,27,28,29,30]. As MFGM is a reservoir of membrane proteins, these proteins were analyzed by research groups using proteomic techniques to identify potential signaling and secretory pathways utilized by the mammary gland [31]. One of the most abundant proteins studied among the MFGM proteins is butyrophilin. This protein has been studied and shown to be involved in modulating the encephalitogenic T-cell response to myelin oligodendrocyte glycoprotein related to human multiple sclerosis [32]. There have been several studies that have utilized the proteomics platform to study and identify MFGM proteins in different milk species including bovine and yak milk [33], cattle [34], goat [35], and horse [36], or to compare protein composition of milk among different breeds in cattle [37] and goat [38].

To the best of our knowledge, we found only one study that has been conducted to characterize the MFGM proteome in camel milk [39]. As it is known that MFGM composition could differ among various breeds of the same animal, the present study targeted to analyze the differential protein composition of MFGM in camel milk produced by two of the Saudi camel breeds, *Camelus dromedaries* (*C. dromedarius*) Safra and Wadha, in order to find the differences in nutritional value depending on the differences in the level of the proteins between them.

## 2. Results

### 2.1. CyDye Fluorescence Labeling and Two-Dimensional Difference in Gel Electrophoresis (2D-DIGE)

**Figure 1:**
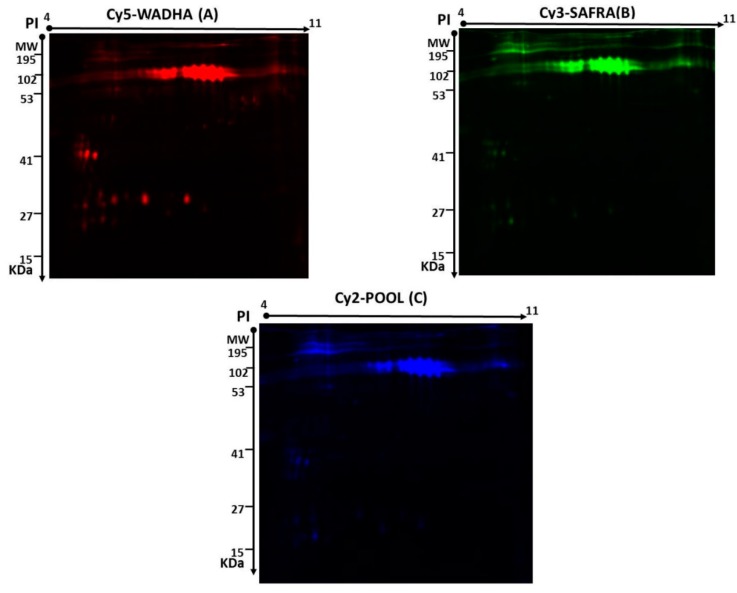
Representative fluorescent protein profiles of a two-dimensional difference in gel electrophoresis (2D-DIGE) containing: milk fat globule membrane (MFGM)-Safra milk samples labeled with Cy3 (**A**), MFGM-Wadha milk samples labeled with Cy5 (**B**), and pooled internal control labeled with Cy2 (**C**).

**Figure 2:**
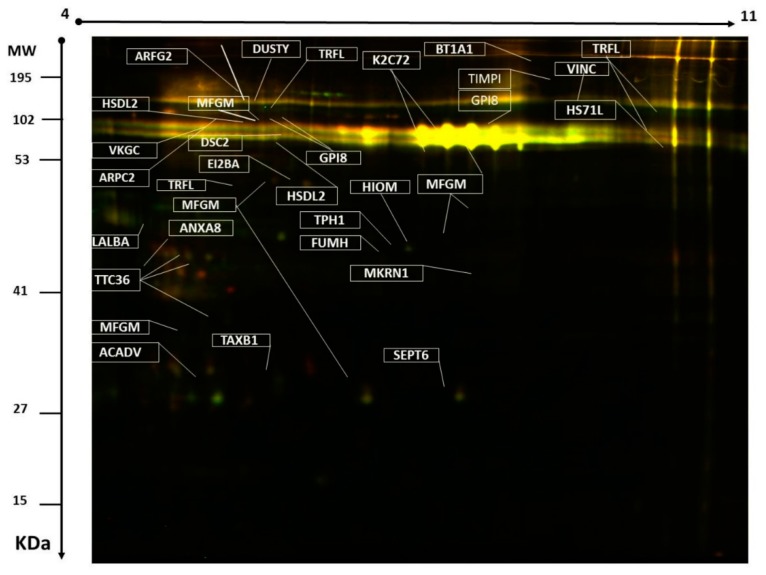
Representative image of the protein spots from milk samples showing the statistically significant differentially expressed spots (ANOVA *p* ≤ 0.05 and fold change ≥1.5, 44 proteins) successfully identified with MALDI-TOF/TOF and labeled with MASCOT IDs.

Two-dimensional (2D)-DIGE was used to assess the differences in the pattern of protein level changes in the MFGM: five Biological Replicates for each breed (Safra and Wadha). Representative fluorescent protein profiles of a 2D-DIGE containing MFGM-Safra sample labeled with Cy3 are shown in Figure 1A; MFGM-Wadha sample labeled with Cy5 (Figure 1B), and a pooled internal control labeled with Cy2 (Figure 1C). Manual validation was performed to exclude false spots. A total of 820 spots were mapped on the gels, of which 60 were significantly different (ANOVA *p* ≤ 0.05 and fold change ≥ 1.5) between the MFGM samples from Safra and Wadha breeds (Figure 2). The gel images show the degree of differential expression of proteins in the merged image, between MFGM-Safra labeled with Cy3 (green) and MFGM-Wadha labeled with Cy5 (red), that are represented as yellow spots. The yellow fluorescent spots represent proteins with the same isoelectric point, molecular weight, and nearly equal fluorescent intensity. The spot patterns were reproducible across all five gels, leading to alignment and further analysis. Cy2-labeling (the internal standard) was included to permit normalization across the complete set of gels and quantitative differential analysis of the protein levels. A total of 60 spots showing a statistical significance between the groups were then manually excised from the preparative gel for protein identification by mass spectrometry.

### 2.2. Mass Spectrometry and Identification of Proteins

**Figure 3:**
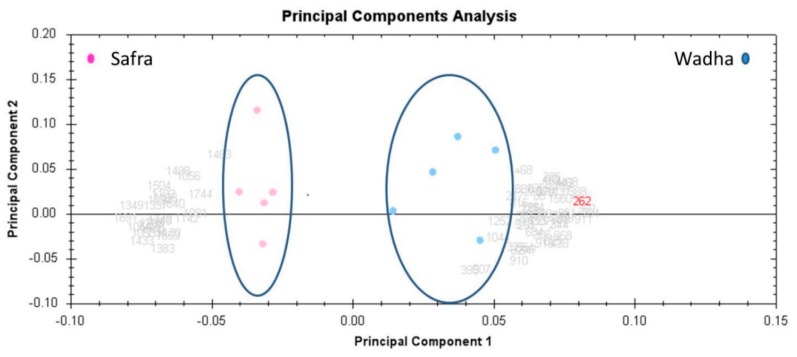
PCA plot of the two first principal components. Both together explained 75.15% of the selected spot’s variability. Colored dots (*n* = 10) and numbers indicate the representation of gels (*n* = 5 of Wadha and *n* = 5 of Safra) and spot proteins number (*n* = 60), respectively.

Peptide mass fingerprint (PMF) successfully identified 44 out of the 60 protein spots excised from the preparative gel. MALDI-TOF mass spectrometry found 25 spots to be unique protein sequences that were matched to entries in the SWISS-PROT database by Mascot with high confidence scores (Table 1, Table S1, Supplementary materials). The sequence coverage of the identified proteins by PMF ranged from 4% to 85%. In a few cases, variants of the same protein were found at several locations on the gel (Table 1, Figure 2). Among the 44 proteins identified, 31 protein spots were up-regulated and 13 down-regulated in MFGM from Safra compared to Wadha breeds (Table 1, Figure 3). The significantly up-regulated proteins included α-lactalbumin lactadherin, annexin a8 hydroxysteroid dehydrogenase-like protein 2 (up 1.6-fold), and GPI-anchor transamidase. The significantly down-regulated proteins included lactotransferrin, vinculin dual serine/threonine and tyrosine protein kinase butyrophilin subfamily 1 member a1 heat shock 70 kDa protein 1-like, acetyl serotonin O-methyl transferase, and ADP-ribosylation factor GTPase-activating protein 2 (a complete list of up-and down-regulated protein has been provided in Table 1, Table S1, Supplementary Materials). Among the identified proteins, lactotransferrin, vinculin, tetratricopeptide repeat protein 36, lactadherin, hydroxysteroid dehydrogenase-like protein 2, GPI-anchor transamidase, keratin, and type II cytoskeletal 72 were found in more than one spot on the gels, which could be explained by post-translational modifications, cleavage by enzymes, or the presence of different protein species. Not all spots of interest could be identified because some proteins were low in abundance and did not yield a sufficiently intense mass of fingerprints; other spots were mixtures of multiple proteins.

### 2.3. PCA and Cluster Analysis

**Figure 4:**
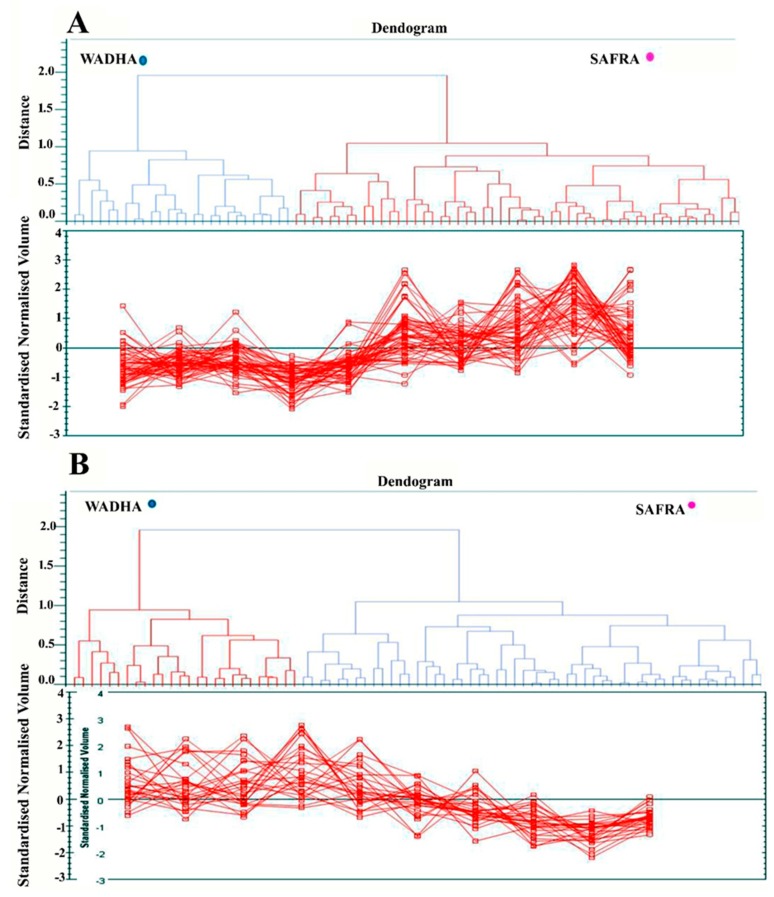
Expression profiles, separated into clusters of expression patterns, indicating the number of spots for each cluster. Each line represents the standardized abundance of a spot across all gels and belongs to one of the clusters generated by hierarchical cluster analysis. (**A**) The spots with increased abundance indicate the 31 proteins up (**B**). The spots with decreased abundance indicate the 13 proteins down in MFGM from Safra breeds compared to Wadha breeds (Progenesis SameSpots).

The unsupervised principal component analysis (PCA) bi-plot of gels and spots (Figure 3) showed distinct gel grouping that agreed with the experimental groups. The PCA was performed using Progenesis SameSpots software to determine and visualize the samples coming from the Safra and the Wadha camel breeds. The PCA was performed on all 60 spot features which exhibited statistically significant (ANOVA *p* < 0.05) changes in abundance, identified by MS. The analyses revealed that the two groups clustered distinctly from one another based on different proteins with a 75.15% score (Figure 3). The differentially abundant spots showed expression pattern clusters according to their abundance patterns based on the hierarchical clustering analysis (Figure 4A,B). The clustering pattern showed the change in the protein intensities for the selected spots between Safra and Wadha MFGM samples were significantly different.

### 2.4. Classification of Proteins Based on Function, Cellular Component, Pathway, and Biological Process

**Figure 5:**
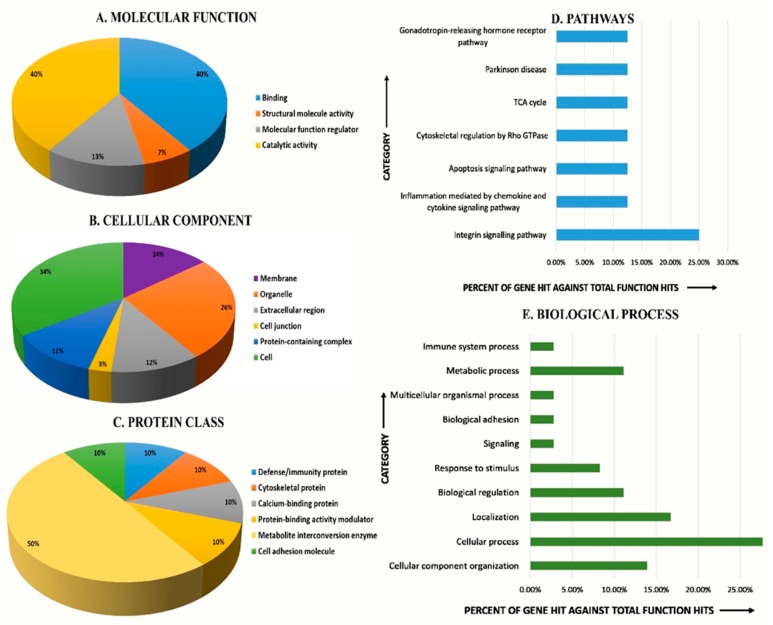
A comparative depiction (%) of identified proteins in MFGM fraction of camel milk in Safra in comparison to the Wadha categorized into groups according to their molecular function (**A**), cellular component (**B**), protein class (**C**), pathways (**D**), and biological process (**E**) using the PANTHER (protein analysis through evolutionary relationships) classification system (http://www.pantherdb.org).

To gain a better understanding of the functional role of the different proteins identified in the data set, and to assess their degree of involvement in the Safra and Wadha MFGM samples, the PANTHER (protein analysis through evolutionary relationships) classification system (http://www.pantherdb.org) was used to classify these proteins based on their known molecular function (Figure 5A), cellular component (Figure 5B), protein class (Figure 5C), pathways (Figure 5D), and biological process (Figure 5E). The proteins identified were found to be involved in catalytic activity (40%) and binding (40%) concerning molecular function, metabolite interconversion enzyme (50%) for protein class, integrin signaling pathway (25%) with respect to pathways associated, and cellular process (27.7%) concerning the biological process. The majority were localized within the cellular component (34%).

### 2.5. Validation by ELISA Immunoassay

**Figure 6:**
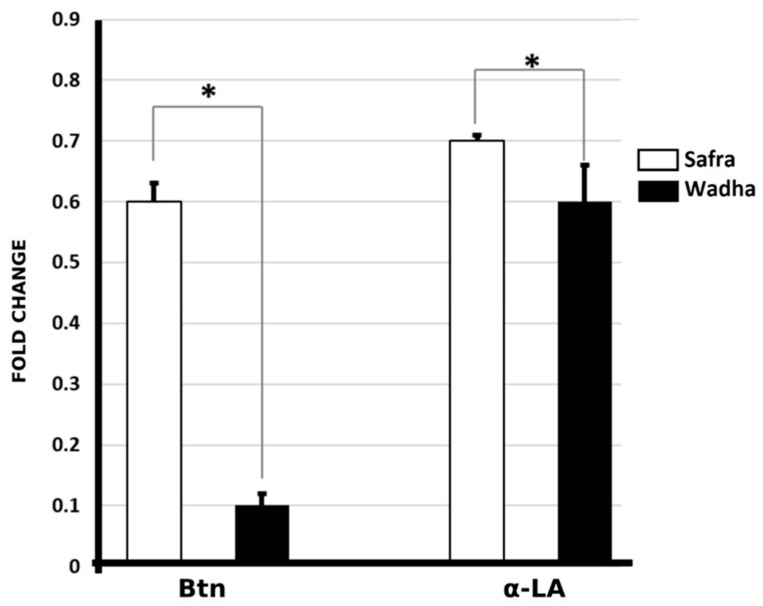
ELISA immunoassay results showed significant differences in the levels of Btn proteins between Safra and Wadha breeds (0.6 ± 0.03 vs. 0.10 ± 0.22, *p* ≤ 0.01) with a fold change of 2.1, up in Safra similar to the DIGE results. This was also seen in the levels of α-LA in Safra and Wadha (0.73 ± 0.01 vs. 0.61 ± 0.06, *p* < 0.01) breeds with a fold change of 1.2 up in Safra breed which was quite similar to the DIGE result. * *p* ≤ 0.01.

The ELISA test was used to validate the proteins in the 15 samples obtained from the Safra and Wadha MFGM samples. Two of the proteins identified in the 2D-DIGE experiments were validated by their respective antibodies (Btn and α-LA). All data were represented as mean ± standard deviation (SD) and *p* < 0.05 and fold change. The statistical results showed a significant difference in the levels of Btn proteins between Safra and Wadha breeds (0.6 ± 0.03 vs. 0.10 ± 0.22, *p* < 0.01) with a fold change of 2.1, up in Safra breed similar to the DIGE results. This was also seen in the levels of α-LA in Safra and Wadha (0.73 ± 0.01 vs. 0.61 ± 0.06, *p* ≤ 0.01) breeds with a fold change of 1.2, up in Safra breed, which was quite similar to the DIGE result (Figure 6).

## 3. Discussion and Conclusions

The MFGM is a complex membrane derived from the mammary epithelial cell which surrounds the fat droplets, mainly triglycerides, in the milk. The membrane is enriched with sphingolipids, cholesterol, glycerophospholipids, and proteins. Some of these proteins are glycosylated which play an important role in biological function during the lactation period [13,25,26] and are diverse in different species. Several reports suggested that the structure and bioactive components of the MFGM play a crucial role in infant gut maturation, neurological development, modulating cholesterol and lipid uptake, immunity [40], and metabolism [41]. Moreover, the MFGM proteins may have an essential role in shaping gut microbial populations, which in turn may provide various health benefits including immune defense [42] and inflammatory diseases early in life [43,44]. Furthermore, MFGM is known to possess anti-carcinogenic potential. For instance, MFGM is capable of inducing apoptosis in human colon cancer cells (HT-29) via the reduction of cell proliferation and increasing the caspase-3 activity, which known as one of the programmed cell death marker, cell cycle arrest in S phase, increasing the apoptotic protein Bax, downregulating the anti-apoptotic protein Bcl-2, and decreasing mitochondrial membrane potential. The data suggested that some of the MFGMs could be used as potential agents for human colon cancer prevention [45,46].

The current study identified 44 proteins of MFGM differentially expressed in Safra and Wadha. Of these proteins, 31 were up-regulated and 13 down-regulated between Safra and Wadha breeds. Among these proteins, two proteins, butyrophilin subfamily 1 member A1 (BTN) and α-lactalbumin (alpha-LA), were validated by ELISA.

Butyrophilin is one of the most abundant MFGM proteins expressed in lactating mammary glands and is a major component of MFGM [47]. BTN is a member of the B7-like proteins family and is encoded by a single gene located telomeric to the HLA complex [48] consisting of two extracellular immunoglobulin domains constant IgC, and variable IgV and a transmembrane region [47]. It is considered as immune system regulator due to is present in immune cells such as lymphocytes, macrophages, monocytes and others [49] and is expressed by thymic stromal cells and inhibits proliferation and cytokine production of stimulated CD4+ and CD8+ T cells [32]. Several studies indicate that BTN influences the clinical outcome of autoimmune responses to myelin oligodendrocyte glycoprotein, an important antigenic target in experimental autoimmune encephalomyelitis and in the etiology of multiple sclerosis [50,51].

We identified numerous spots relating to the protein α-lactalbumin. It is known to form peptides, some of which have biological activities during digestion. This protein has a specific binding site for calcium and other for essential trace elements including iron and zinc [52]. Peptides from α-lactalbumin can facilitate the uptake of essential micronutrients by the intestinal mucosal cell [53]. Several studies indicate that humans, as well as α-lactalbumin from many primate milks, are lethal to tumor cells, as they result in death to the cancer cells through apoptosis without affecting the healthy cells [54,55,56,57,58]. In humans, α-lactalbumin can reduce the occurrence of cancer in breastfeeding children by removing tumor cells from the gut of the neonate by inducing apoptosis in tumor cells but spares healthy cells [55,56]. Meanwhile, camel α-lactalbumin was shown to be more disordered and possessed stronger aggregation propensities [52,59]. The structural differences between the camel and bovine α-lactalbumin were preserved and in some cases increased in their oleic acid complexes, which mainly increased in its lethality against tumor cells [52]. Our data shows that α-lactalbumin is overexpressed in the Safra breed with a 2.8-fold change in contrast to the Wadha breed.

Other proteins identified in our study include tetratricopeptide repeat protein 36 (TTC36 also named HBP21) which is capable of inducing apoptosis via the translocation of Bax from cytoplasm-to-mitochondria which leads to increase the cleaved of caspase-3, caspase-9, and poly (ADP ribose) polymerase tumor cells including hepatocellular carcinoma [60]. Moreover, HBP21 might be involved in the inhibition of progression and metastasis of breast cancer cells [61].

MFGM in camel milk not only has nutrient value but also involves cancer cell elimination properties. The milk obtained from the Wadha breeds showed increased spots relating to lactoferrin, vinculin, dual serine/threonine and tyrosine protein kinase (DUSTY_BOVIN), heat shock 70-kDa protein-1-like (HSPA1L), and Tax1 (human T-cell leukemia virus type I)-binding protein 1 (TAX1BP1). Lactoferrin (Lf, also known as lactotransferrin) is an iron-binding glycoprotein that belongs to the transferrin protein family [62] and has several biological functions including anti-bacterial, anti-viral, anti-inflammatory, and anti-tumor [8,10]. This protein is highly expressed in Wadha breeds compared to Safra breeds.

The protein vinculin has also been shown to be protective against different cancers and loss of vinculin has been shown to be involved in the development of various cancers including breast cancer [63], squamous carcinoma [64], and colorectal cancer (CRC) [65]. This implies that vinculin may have anti-tumor effects. Our data showed that vinculin is overexpressed in Wadha breeds in contrast to Safra breeds. DUSTY_BOVIN is known as RIP5 involves inactivation of p38, JNK, NF-κB, and induces both caspase-dependent apoptosis and caspase-independent cell death [66]. The current results reveal that RIP5 is overexpressed with a 2.5-fold change in Wadha compared to Safra milk. HSPA1L acts as a cellular prion protein stabilizer in CRC progression tumorigenicity in vivo. HSPA1L expression stabilized hypoxia-inducible factor-1α (HIF-1α) protein and promoted the cellular prion protein (PrPC) accumulation through the HSPA1L/HIF-1α/GP78 axis [67]. The presented results showed that the HSPA1L is expressed with a 2.4-fold change in Wadha contrast to Safra milk. Heat shock binding protein 21 (HBP21), also known as Tetratricopeptide Repeat Domain 36 (TTC36), is known to inhibit tumor cell growth rate and tumor formation in nude mice when it was transfected into hepatocellular carcinoma. HBP21 can inhibit the interaction of HSP70 and Bax. As a result, Bax will be translocated cytoplasm-to-mitochondria and then induce apoptosis through the releasing cytochrome c and activating caspase-3 and caspase-9 [60]. Another study in breast cancer showed that the interaction of Hsp70 and HBP21 is might capable of the inhibition of progression and metastasis of this cancer [61]. In our data, the expression of HBP21 was overexpressed in Safra with a 2.6-fold change compared to Wadha milk.

Not only a complex of human α-lactalbumin and oleic acid (HAMLET), but bovine (BAMLET) and camel (CAMLET) acts as a potent and differential anticancer agent [57]. BAMLET and CAMELT are capable of activation of the caspase-independent lysosomal cell death pathway in highly apoptosis-resistant tumor cells [57,58,68]. Furthermore, HAMLET can trigger apoptosis of bladder cancer cells in vivo bladder [69], human glioblastoma [70], and not affecting health cells even when high concentrations of HAMLET is used. Besides, Li X. et al. demonstrated that α-lactalbumin promoters might be able to regulate the replication of adenovirus to target hormone-independent breast cancers. As a result, the α-lactalbumin promoter might be used to create a novel therapeutic target for hormone-independent breast cancer [71]. Our data indicated that α-lactalbumin is overexpressed with a 2.8-fold change in Safra in contrast with Wadha milk. Tax1 (human T-cell leukemia virus type I)-binding protein 1 (TAX1BP1) is collaborating with A20 binding inhibitor of NF-κB 1 (ABIN1). This leads to the negative regulator of antiviral signaling [72]. Additionally, TAX1BP1 plays a crucial role in protecting cells from virus-induced apoptosis [73]. Our data revealed that the expression of TAX1BP1 is 2.5-fold in Safra higher compared to Wadha milk. This may indicate that drinking Wadha milk might provide more immunity and protection against infections than drinking Safra milk. Identification of all of these proteins indicates that drinking Wadha milk may have more anti-tumor activity compared to Safra milk.

## 4. Materials and Methods

### 4.1. Camel Milk Samples Collection

Camel milk (250 mL) was collected from 10–15 healthy camels of the two breeds (Wadha and Safra) reared in the same area with similar conditions of nutrition and stage of lactation. Characteristics of the two Saudi Arabian camel breeds Safra and Wadha and the respective milk samples obtained from them are shown in Table S2. All samples were taken from the farms in the Jeddah city area, Saudi Arabia. The samples were collected in clean sterilized 0.5 L bottles and transferred immediately to the lab in the temperature-controlled icebox. On arrival to the lab, 0.1% sodium azide was added to each sample and gently mixed together to prevent microbial growth and contamination of samples. The samples were then aliquoted in 50 mL labeled falcon tubes and stored in −20 °C freezer until further analysis

### 4.2. Preparation of MFGM Samples

Five samples (50 mL) of camel milk from the two breeds (Safra and Wadha) were taken. The temperature of samples was controlled at 2–8 °C during the procedures according to Saadaoui et al. (2013) and Pisanu et al. (2012) [39,74] with minor modifications. Briefly, the samples were centrifuged at 4000 rpm for 35 min to separate the creamy layer which was transferred to a new 50 mL falcon. The milk fat quantity was measured to maintain and 1M PBS (4.3 mM Na_2_HPO_4_, 2.7 mM KCl, 1.8 mM KH_2_PO_4_, 137 mM NaCl and pH adjusted to 7.4) was added 5 times and kept in a 37 °C PBS water bath for 20 min with regular shaking to melt the milk fat and separate cluster of sample. The sample was further centrifuged at 4000× *g* rpm for 35 min twice with PBS and one time with MilliQ water to remove residual casein and whey proteins. After each washing step, the milk fat sample was transferred into a new falcon tube. At the end of the washing process, the sample was kept at 2–8 °C overnight. The next day the samples were mixed with sterile plastic beads using a vortex mixer at 60 hertz (Hz) for 3 min for homogenization. The samples were warmed up to 37 °C using a water bath for 10 min and centrifuged at 4000× *g* rpm for 30 min to separate the MFGM from the top oily yellow fat layer which was further discarded. The white pellets remaining in the tube were the MFGM component, they were weighed and stored at −20 °C freezer until further analysis [39,74].

### 4.3. Extraction of MFGM Proteins

The extraction of MFGM was done as described previously [75,76] with some modifications. Briefly, the MFGM pellets were incubated with lysis buffer (pH 8.8, 30 Mm Tris buffer containing 7 M urea, 2 M thiourea, 2% Chaps, 1× protease inhibitor mix), for 1 h with periodic vortexing. Samples were then incubated in a water bath for 5 min at 95 °C and centrifuged at 12,000× *g* for 15 min. Then, 50 Mm dithiothreitol (DTT) was next added and the lysates were centrifuged again (20,000× *g*, 40 min, 4 °C) to obtain the solubilized proteins. The solubilized proteins in the supernatant were collected and precipitated using a 2D clean-up kit according to the manufacturer’s protocol (GE Healthcare, USA) and the pH of the samples was adjusted to 8.5 using NaOH (100 mM) before 2D-DIGE analysis. The protein concentration of each sample was then determined in triplicate using the 2D-Quant Kit (GE Healthcare, Chicago, IL, USA).

### 4.4. D-DIGE and MALDI-TOF Analysis

#### 4.4.1. Sample Labeling with Cyanine Dyes

Proteins were labeled according to the manufacturer (GE Healthcare, Chicago, IL, USA). Briefly, 50 µg of milk MFGM-Wadha (*n* = 5) and milk MFGM-safra (*n* = 5) protein extract samples were minimally labeled with CyDye™ DIGE Samples and incubated on ice for 30 min in the dark. The labeling reaction was terminated by adding 1 μL of 10 mM lysine. Each MFGM samples from Safra and Wadha were covalently labeled with a fluorophore, either Cy3 or Cy5. A mixture of an equal amount of all samples was pooled, labeled with Cy2, and used as an internal standard. A dye switching strategy was applied during labeling to avoid dye-specific bias (Table S3, Supplementary materials).

#### 4.4.2. One- and Two-Dimensional Difference Electrophoresis (2D-DIGE)

One-dimensional analytical gel electrophoresis was performed using Immobiline Dry Strips (24 cm, pH 3-11; GE Healthcare, Sweden) and then the IPG strips were equilibrated, first with dithiothreitol (DTT) (15 min, RT, gentle stirring, 5 mM Tris–HCl, pH 8.8, 6M urea, 30% glycerol, 2% SDS 65 mM). Strips were then equilibrated for 15 min in the same solution containing 250 mM iodoacetamide (IAA). followed by second-dimension sodium dodecyl sulfate polyacrylamide gel electrophoresis (SDS-PAGE) performed on 12.5% fixed gels using an Ettan Dalt Six device (GE Healthcare, Sweden) were carried out as previously described by Alfadda et al. [77]. Further, the 2D-DIGE gels were scanned on the Typhoon 9410 scanner using excitation/emission wavelengths specific for Cy2 (488/520 nm), Cy3 (532/580 nm), and Cy5 (633/670 nm).

#### 4.4.3. Statistical Analysis

The 2D-DIGE gel images were uploaded into Progenesis “SameSpots” software (Nonlinear Dynamics UK) and were analyzed applying an automated spot detection method. The analysis compared the Safra and Wadha MFGM samples. Although the automatic analysis was performed to detect all the spots across all the 5 gels, each selected spot was verified and manually edited wherever necessary. Normalized volumes were used to identify spots that were differentially expressed. A cut-off ratio ≥ of 1.5-fold was considered. ANOVA was used to calculate statistically significant differences between groups. *p* < 0.05 was considered statistically significant.

#### 4.4.4. Protein Identification by MALDI-TOF MS

Coomassie-stained gel spots from the preparatory gel were washed and digested according to previously described methods [76,77,78]. The mixture of tryptic peptides (1 μL) derived from each protein was spotted onto a MALDI target (384 Anchorchip MTP 800 μm Anchorchip; Bruker Daltonik, Bremen, Germany). MALDI-MS(/MS) spectra were obtained using an ultrafleXtreme time-of-flight (TOF) mass spectrometer equipped with a LIFT-MS/MS device (Bruker Daltonics) instrument as described [75,79] at a reflector voltage of 21 kV and the detector voltage of 17 kV. Peptide mass fingerprints (PMF) were calibrated against a standard (peptide calibration standard II, Bruker Daltonics). The PMFs were assessed using Flex Analysis software (version 2.4, Bruker Daltonics). MS data were interpreted by using BioTools version 3.2 (Bruker Daltonics). The peptide masses were searched against the Mascot search algorithm (version 2.0.04 updated 09/05/2019; Matrix Science Ltd., London, UK). Identified proteins were screened for a Mascot score of higher than 56 and *p* < 0.05.

### 4.5. Gene Ontology Enrichment Analysis

The accession ID obtained from the UniProt gene ontology system for annotation was further uploaded in PANTHER (protein analysis through evolutionary relationships) classification system (http://www.pantherdb.org) to classify the identified proteins into different functional categories. The results for molecular function, cellular components, protein class, biological process, and pathways were exported and pie charts and bar graphs were designed using Microsoft Excel. The graphs generated depict different categories compared to percent of gene hit against total function hits (from data obtained from PANTHER).

### 4.6. ELISA Immunoassay Validation for Selected MFGM Proteins

The sandwich ELISA was carried out using the previously described method by El-Fakharany et al. (2017) with modification [1]. Briefly, high binding ELISA microplates were coated with 50 µL of coating solution in duplicate manner (coating buffer; 3.03 g of Na_2_CO_3_ mixed with 6.0 g of NaHCO_3_ to make 1 L, pH 9.6) and MFGM protein samples from Safra and Wadha were added at a concentration of 2.2 µg/mL. The plates were sealed and incubated for a period of 3 h at 37 °C in a controlled incubator followed by washing steps (3 times with 10 mMTris pH 8.8, 0.01% Tween 20 and 0.5% gelatin, using automated microplate washer (BIO-TEK, ELx50)). The plates were inverted and blotted on clean adsorbing paper to remove any residual washing solution. Afterwards, 50 µL blocking buffer (2% gelatin and 0.1% Tween 20 in 10 mMTris) was added to each well in the ELISA microplates, followed by second incubation for 1 h at 37 °C. The 2 primary monoclonal antibodies (Btn, and α-LA) obtained from DSHB (created by the Eunice Kennedy Shriver National Institute of Child Health and Human Development of the National Institutes of Health (NIH) and maintained at The University of Iowa, Department of Biology, Iowa City, IA 52242, USA) were used. Then, 50 µL of these monoclonal antibodies were added in duplicate manner to each sample in the ELISA microplates at a concentration of 0.5 µg/mL, and incubated at 37 °C for 2 h. The plates were washed 3 times and 50 µL of secondary antibodies (goat anti-mouse IgG H&L (HRP) ab97023, Abcam and goat anti-rabbit IgG H&L (HRP) 31460, Thermo Fisher Scientific, United States) were added at a concentration of 1:50,000 after diluting with wash buffer. The plates were incubated for 1 h at 37 °C and 50 µL of TMB substrate was added and incubated in dark ambient temperature for 20 min. Lastly, 50 µL of stop solution (1M H_2_SO_4_) was added and the plates were read within 5 min by a microplate reader (BioTek, ELx800) at 450 nm wavelength [1]. All data were calculated and represented as mean ± standard deviation (SD).

### 4.7. Hierarchical Clustering and Statistical Analysis

Proteins were grouped by their expression patterns using hierarchical clustering by a commercial Progenesis “SameSpots” software (Nonlinear Dynamics, UK). This unsupervised, multivariate analysis provides a true snapshot of protein activity across an experiment and shows the expression profiles for all the significant proteins, increased or decreased in abundance, across all the samples. The results are shown in an interactive dendrogram tree where similar expression profiles cluster together. Data can be explored at group level on the dendrogram, and the corresponding expression profiles (http://www.nonlinear.com/products/progenesis/stats/overview/).

Statistical analysis of experimental data was performed using SPSS (IBM Corp. Released 2012. IBM SPSS Statistics for Windows, Version 21.0. Armonk, NY: IBM Corp., Armonk, NY, USA).

## Figures and Tables

**Table 1:** Identified proteins with changes in abundance between MFGM-Safra and Wadha breed samples. The average ratio values along with their corresponding levels of fold changes and one-way ANOVA (*p* < 0.05) using 2D-DIGE. (Analysis type: MALDI-TOF; database: SwissProt; taxonomy: Other Mammalian).

Spot Number	Accession Number	Mascot ID	Protein Name	Function	Fold Change S/W
**1346**	P00710	LALBA_CAMDR	A-lactalbumin	-	2.8
**1583**	Q3SZV0	TTC36_BOVIN	Tetratricopeptide repeat protein 36	-	2.6
**1662**	Q2KJE0	TAXB1_BOVIN	Tax1-binding protein 1 homolog	Binding Protein	2.5
**1613**	P79385	MFGE8_PIG	Lactadherin	Cell adhesion	2.3
**1004**	A4FUZ6	HSDL2_BOVIN	Hydroxysteroid dehydrogenase-like protein 2	Enzyme	2
**1001**	Q95114	MFGE8_BOVIN	Lactadherin	Cell adhesion	2
**1648**	P48818	ACADV_BOVIN	Very long-chain specific acyl-CoA dehydrogenase, mitochondrial	Enzyme	1.6
**1407**	Q95L54	ANXA8_BOVIN	Annexin A8	Cell membrane	1.6
**1155**	A4FUZ6	HSDL2_BOVIN	Hydroxysteroid dehydrogenase-like protein 2	Enzyme	1.6
**1078**	P79385	MFGE8_PIG	Lactadherin	Cell adhesion	1.6
**1676**	Q3SZN0	SEPT6_BOVIN	Septin-6	Cell membrane	1.5
**1460**	Q9TT91	MKRN1_MACEU	E3 ubiquitin-protein ligase makorin-1	Enzyme	1.5
**1429**	P10173	FUMH_PIG	Fumarate hydratase, mitochondrial	Enzyme	1.5
**1416**	Q3SZV0	TTC36_BOVIN	Tetratricopeptide repeat protein 36	-	1.5
**1384**	P79385	MFGM_PIG	Lactadherin	Cell adhesion	1.5
**1373**	P17290	TPH1_RABIT	Tryptophan 5-hydroxylase 1	Enzyme	1.5
**1235**	P79385	MFGE8_PIG	Lactadherin	Cell adhesion	1.5
**1181**	P79385	MFGE8_PIG	Lactadherin	Cell adhesion	1.5
**1072**	P33545	DSC2_BOVIN	Desmocollin-2	Cell membrane	1.5
**1066**	Q3MHZ7	GPI8_BOVIN	GPI-anchor transamidase	Enzyme	1.5
**1063**	P79385	MFGE8_PIG	Lactadherin	Cell membrane	1.5
**1056**	Q148H8	K2C72_BOVIN	Keratin, type II cytoskeletal 72	Cell membrane	1.5
**1054**	P26234	VINC_PIG	Vinculin	Cell membrane	1.5
**1040**	Q3MHR7	ARPC2_BOVIN	Actin-related protein 2/3 complex subunit 2	Cell membrane	1.5
**1039**	Q9MYY3	VKGC_DELLE	Vitamin K-dependent gamma-carboxylase	Enzyme	1.5
**1026**	Q148H8	K2C72_BOVIN	Keratin, type II cytoskeletal 72	Cell membrane	1.5
**1011**	Q3MHZ7	GPI8_BOVIN	GPI-anchor transamidase	Enzyme	1.5
**1009**	Q3MHZ7	GPI8_BOVIN	GPI-anchor transamidase	Enzyme	1.5
**1005**	P79385	MFGE8_PIG	Lactadherin	Cell adhesion	1.5
**396**	P79385	MFGE8_PIG	Lactadherin	Cell adhesion	1.5
**1639**	P79385	MFGE8_PIG	Lactadherin	Cell adhesion	1.4
**898**	P20414	TIMPI_BOVIN	Metalloproteinase inhibitor 1	Enzyme	−1.4
**1423**	Q3SZV0	TTC36_BOVIN	Tetratricopeptide repeat protein 36	Binding Protein	−1.5
**1210**	Q0IIF2	EI2BA_BOVIN	Translation initiation factor eIF-2B subunit alpha	Binding Protein	−1.5
**1225**	Q9TUM0	TRFL_CAMDR	Lactotransferrin	Enzyme	−1.6
**699**	A1L520	ARFG2_BOVIN	ADP-ribosylation factor GTPase-activating protein 2	Binding Protein	−1.9
**358**	Q9TUM0	TRFL_CAMDR	Lactotransferrin	Enzyme	−1.9
**858**	P10950	HIOM_BOVIN	Acetyls erotonin O-methyl transferase	Enzyme	−2.2
**1103**	P0CB32	HS71L_BOVIN	Heat shock 70 kDa protein 1-like	Immune system	−2.4
**871**	P18892	BT1A1_BOVIN	Butyrophilin subfamily 1 member A1	Enzyme	−2.5
**821**	Q4TVR5	DUSTY_BOVIN	Dual serine/threonine and tyrosine protein kinase	Enzyme	−2.5
**916**	P26234	VINC_PIG	Vinculin	Cell membrane	−4.8
**917**	Q9TUM0	TRFL_CAMDR	Lactotransferrin	Enzyme	−5.8
**864**	Q9TUM0	TRFL_CAMDR	Lactotransferrin	Enzyme	−15.8

## Supplementary Materials

The following are available online, Table S1: Mass spectrometry list of significant differentially abundant proteins, Table S2: Experimental design.
