# Supplementary material for: Comparative Analysis of Milk Fat Globular Membrane (MFGM) Proteome between Saudi Arabia Camelus dromedary Safra and Wadha Breeds
Source: Molecules. 2020 May 4;25(9):2146. doi: 10.3390/molecules25092146 (PMC7249027; doi:10.3390/molecules25092146)
Supplement: Supplementary file 1 [file molecules-25-02146-s001.pdf]

Supplementary material

Table 1. List of the differentially abundant significant MFGM proteins identified in the camel milk between Safra and Wadha breed using 2D-DIGE mass spectrometry. Protein name, accession numbers (according to Uniprot database), pI values (according to SWISSPROT), protein MW, MS % coverage and MASCOT scores database are listed.

Spot Number	Accession Number[a]	Protein name	Pi[b]	MW[c]	Cov%[d]	Score[e]
**358**	Q9TUM0	Lactotransferrin	8.66	79158	28	123
**396**	P79385	Lactadherin	6.18	46722	28	166
**699**	A1L520	ADP-ribosylation factor GTPase-activating protein 2	8.07	57065	17	57
**821**	Q4TVR5	Dual serine/threonine and tyrosine protein kinase	6.4	106163	17	57
**858**	P10950	Acetyls erotonin O-methyl transferase	5.64	38470	24	56
**864**	Q9TUM0	Lactotransferrin	8.66	79158	36	125
**871**	P18892	Butyrophilin subfamily 1 member A1	5.11	59923	25	99
**898**	P20414	Metalloproteinase inhibitor 1	8.47	23700	29	57
**916**	P26234	Vinculin	5.62	124437	12	57
**917**	Q9TUM0	Lactotransferrin	8.66	79158	42	173
**1001**	P79385	Lactadherin	6.18	46722	10	170
**1004**	A4FUZ6	Hydroxysteroid dehydrogenase-like protein 2	8.46	45519	23	61
**1005**	P79385	Lactadherin	6.18	46722	16	60
**1009**	Q3MHZ7	GPI-anchor transamidase	5.8	45486	22	58
**1011**	Q3MHZ7	GPI-anchor transamidase	5.8	45486	22	58
**1026**	Q148H8	Keratin, type II cytoskeletal 72	7.86	57578	30	69
**1039**	Q9MYY3	Vitamin K-dependent gamma-carboxylase	8.47	88024	15	58
**1040**	Q3MHR7	Actin-related protein 2/3 complex subunit 2	6.84	34442	29	58
**1054**	P26234	Vinculin	5.62	124437	13	62
**1056**	Q148H8	Keratin, type II cytoskeletal 72	7.86	57578	18	70

Spot Number	Accession Number[a]	Protein name	Pi[b]	MW[c]	Cov%[d]	Score[e]
**1063**	P79385	Lactadherin	6.18	46722	27	57
**1066**	Q3MHZ7	GPI-anchor transamidase	5.8	45486	22	58
**1072**	P33545	Desmocollin-2	5.07	97069	16	57
**1078**	P79385	Lactadherin	6.18	46722	33	72
**1103**	P0CB32	Heat shock 70 kDa protein 1-like	5.89	70744	12	59
**1155**	A4FUZ6	Hydroxysteroid dehydrogenase-like protein 2	8.46	45519	19	67
**1181**	P79385	Lactadherin	6.18	46722	16	72
**1210**	Q0IIF2	Translation initiation factor eIF-2B subunit alpha	6.90	34134	39	58
**1225**	Q9TUM0	Lactotransferrin	8.66	79158	42	163
**1235**	P79385	Lactadherin	6.18	46722	22	57
**1346**	P00710	Alpha-lactalbumin	5	14877	38	66
**1373**	P17290	Tryptophan 5-hydroxylase 1	6.95	51599	31	63
**1384**	P79385	Lactadherin	6.18	46722	26	57
**1407**	Q95L54	Annexin A8	5.3	36992	28	59
**1416**	Q3SZV0	Tetratricopeptide repeat protein 36	5.09	20660	23	57
**1423**	Q3SZV0	Tetratricopeptide repeat protein 36	5.09	20660	23	60
**1429**	P10173	Fumarate hydratase, mitochondrial	7.01	50149	14	57
**1460**	Q9TT91	E3 ubiquitin-protein ligase makorin-1	5.16	54296	10	58
**1583**	Q3SZV0	Tetratricopeptide repeat protein 36	5.09	20660	23	60
**1613**	P79385	Lactadherin	6.18	46722	46	129
**1639**	P79385	Lactadherin	6.18	46722	23	57
**1648**	P48818	Very long-chain specific acyl-CoA dehydrogenase, mitochondrial	8.74	71003	16	58
**1662**	Q2KJE0	Tax1-binding protein 1 homolog	5.34	94855	15	60
**1676**	Q3SZN0	Septin-6	6.35	49080	21	58

[a] Protein accession numbers based on UNIPROT Database.

[b] Theoretical isoelectric point.

[c] Theoretical relative mass.

[d] MASCOT coverage

[e] MASCOT score

Table S2: Characteristics of the two Saudi Arabian camel breeds Safra and Wadha and the respective milk samples obtained from them.

**Variables**	**WADHA**	**SAFRA**
Number of sample	5	5
Age (years)	7.5 ± 1.67	6.9 ± 1.94
Quantity of milk/ week (L)	40 ± 3.53	22 ± 10.36
Age of calf (month)	13 ± 0.7	10 ± 2.16
No. of delivery	2 ± 0.7	1 ± 0,5
Season	Spring	Spring
Type of Food	Sugar cane<br>Bread<br>Alfalfa	Sugar cane<br>Bread<br>Alfalfa
Location	Alharazat<br>Alsawarekh province Jeddah city	Alharazat<br>Alsawarekh province Jeddah city
No. of males in the flock	1.6 ± 0.54	1 ± 0
Time and Date of Sampling	Early morning<br>2-3-2017	Early morning<br>5-3-2017

Table S3: Experimental design: The 10 camel milk samples were run on 5 analytical 2D-DIGE gels, labeled randomly with Cy3 and Cy5, and a pooled sample, used as an internal standard, was labeled with Cy2.

**Gel number**	**Cy3**	**Cy5**	**Cy2**
G1	SAFRA 1	WADHA1	Pooled Sample
G2	WADHA2	SAFRA2	Pooled Sample
G3	SAFRA3	WADHA3	Pooled Sample
G4	WADHA4	SAFRA4	Pooled Sample
G5	SAFRA5	WADHA5	Pooled Sample
